# Supplementary material for: Spreading depression as an innate antiseizure mechanism
Source: Nat Commun. 2021 Apr 13;12:2206. doi: 10.1038/s41467-021-22464-x (PMC8044138; doi:10.1038/s41467-021-22464-x)
Supplement: Supplementary file 1 — Supplementary Information [file 41467_2021_22464_MOESM1_ESM.pdf]

Supplementary Information for

SPREADING DEPRESSION AS AN INNATE ANTISEIZURE MECHANISM

Isra Tamim[1,2], David Y. Chung[1,3], Andreia Lopes de Morais[1], Inge C.M. Loonen[1], Tao Qin[1], Amrit Misra[3], Frieder Schlunk[2], Matthias Endres[2], Steven J. Schiff[4], and Cenk Ayata[1,3]*

1 Neurovascular Research Unit, Department of Radiology, Massachusetts General Hospital, Harvard Medical School

2 Charité – Universitätsmedizin Berlin, Klinik und Hochschulambulanz für Neurologie und Centrum für Schlaganfallforschung Berlin (CSB)

3 Department of Neurology, Massachusetts General Hospital, Harvard Medical School

4 Center for Neural Engineering, Departments of Engineering Science and Mechanics, Neurosurgery, and Physics, The Pennsylvania State University

Corresponding Author:
Cenk Ayata, M.D., Ph.D.
149 13th Street, Room 6408, Charlestown, MA 02129, USA
Office: (617) 726 0821; Cell: (617) 543 5442; Fax: (617) 726 2547; email: CAYATA@mgh.harvard.edu

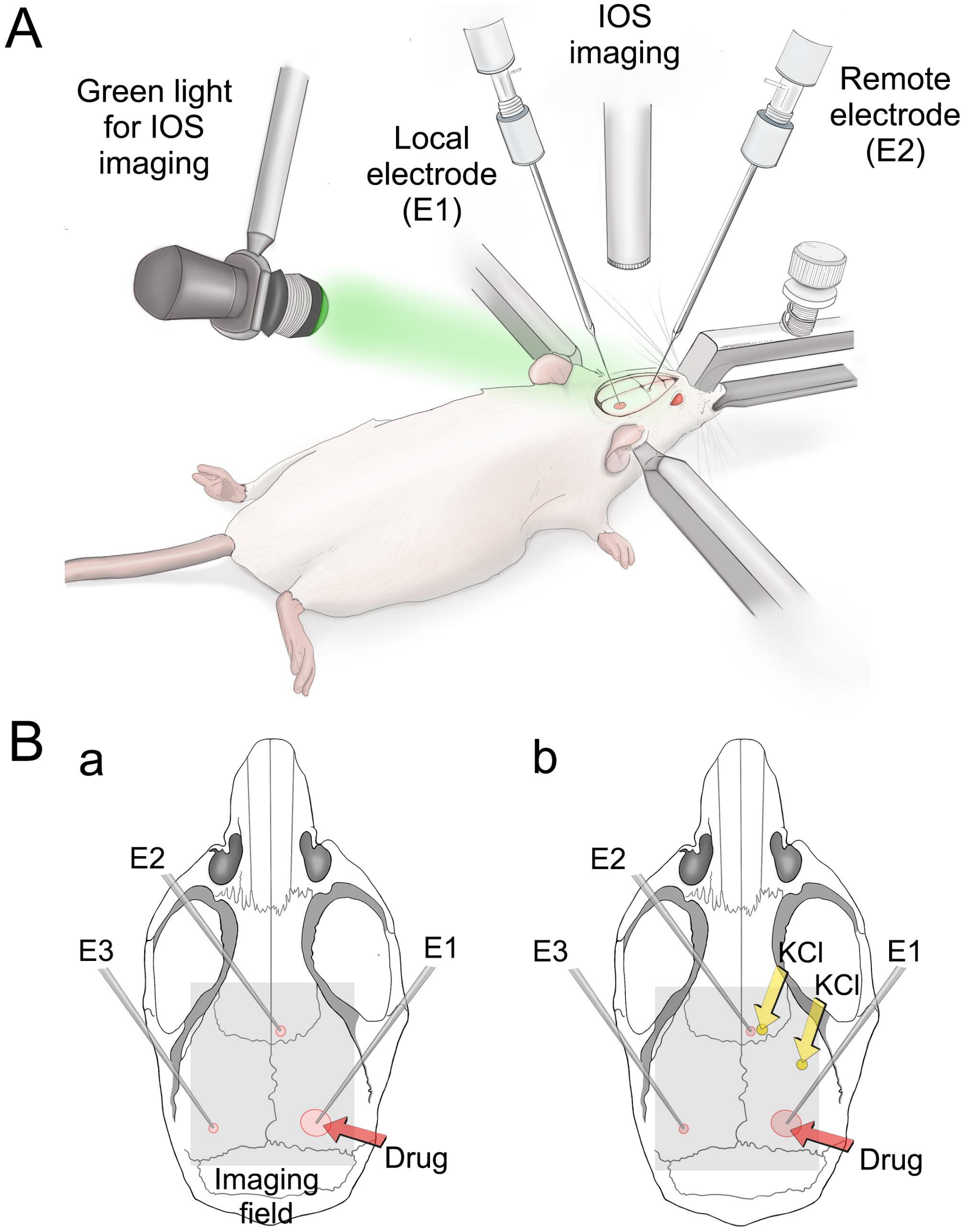

Supplementary Figure 1. Experimental setup

(A) Basic experimental setup consisted of light source (530 nm green LED shown) to illuminate the skull, USB pen camera to image the field at 1Hz through intact skull, and 2-3 epidural glass micropipettes to record electrocorticogram (ECoG) and slow (DC) potential. **(B)** Seizures were generated by topical application of epileptogenic agents onto an occipital window consisting of thinned skull with a small crack to insert the epidural electrode and enhance drug diffusion (2 mm diameter, 3 mm lateral and 2 mm anterior from lambda, red arrow). Left and right panels show the most common electrode configurations (E1-E3) to capture seizure generalization and SD occurrence. E1, local electrode over the seizure focus; E2, ipsilateral remote electrode (1.5 mm lateral, 1.5 mm anterior from bregma); E3, contralateral remote electrode in homotopic to seizure focus. Grey shade shows IOS imaging field. Right panel also shows the sites of topical KCl application onto thinned skull (yellow arrows) to trigger SD. In experiments with an E2, KCl was applied between the two recording sites (lower arrow), whereas in experiments without an E2, KCl was applied onto the anterior window (upper arrow).

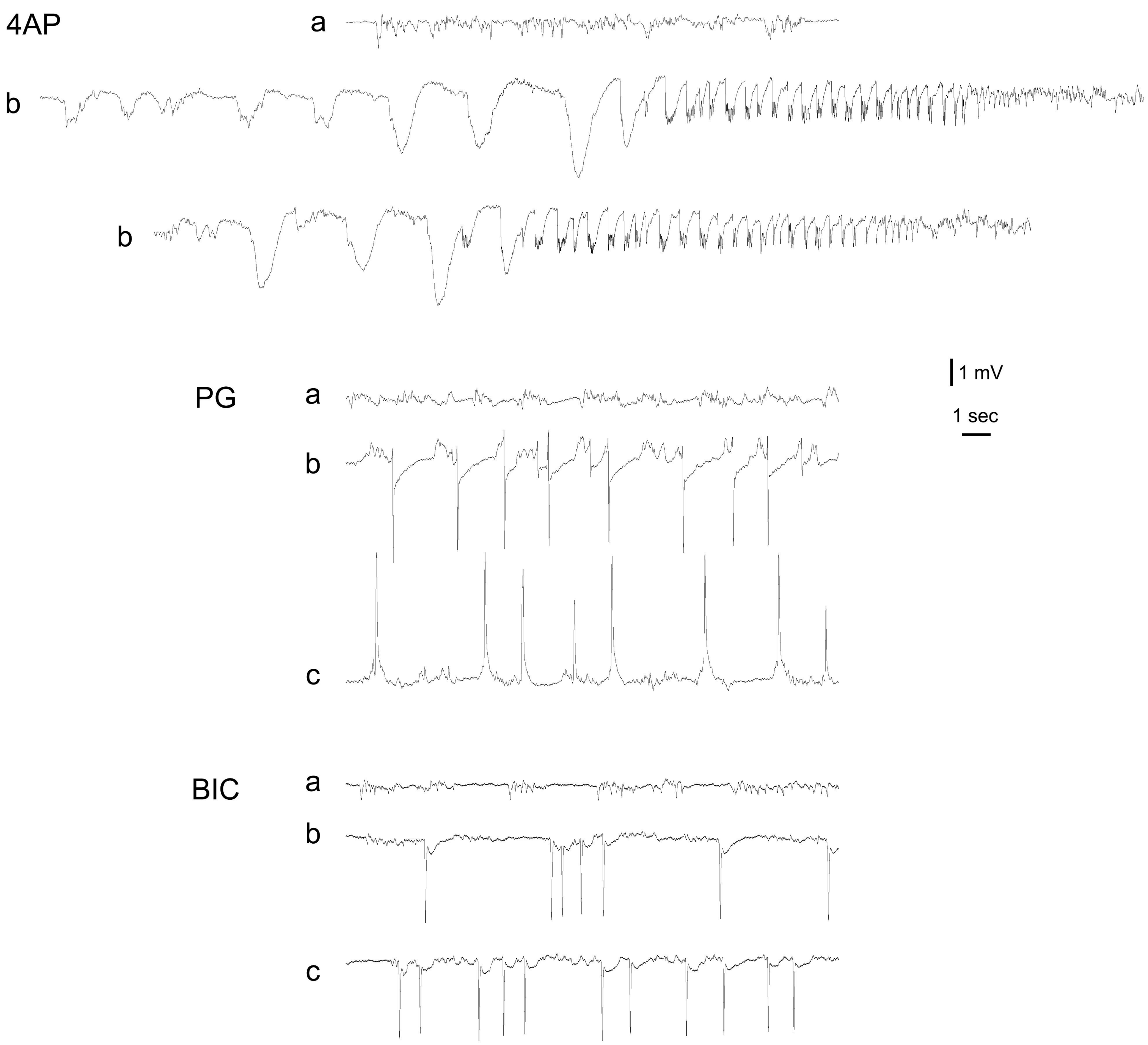

Supplementary Figure 2. Seizure characteristics after topical 4AP, PG or BIC application.
Electrocorticographic changes after topical 4AP, PG or BIC application shown on an expanded time scale. Source data are provided as a Source Data file.

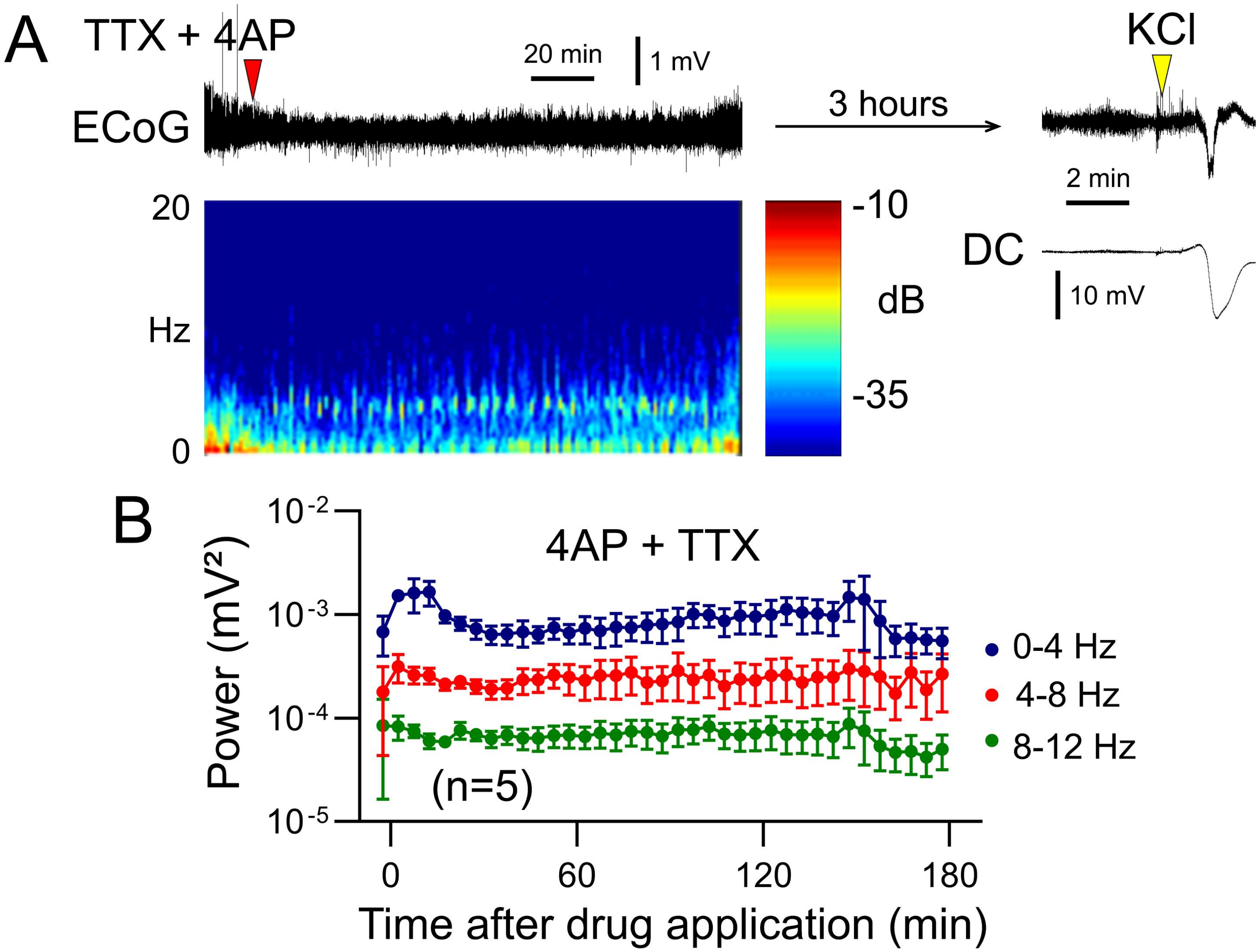

Supplementary Figure 3. Inhibition by tetrodotoxin confirms seizures as the immediate trigger for SD.
Pretreatment of cortex with TTX (30 μM) 30 minutes before 4AP application prevented seizures, as shown in a representative ECoG tracing and time–frequency-power spectrum **(A)**, and the group average of ECoG power over time **(B)**. As a result, no SD was detected in any of the experiments after TTX pretreatment. At the end of each experiment, we confirmed that TTX did not directly block SDs by inducing an SD using topical KCl application at the ipsilateral remote site and detecting it in the 4AP application site (shown in upper right). Data are mean±SEM. Source data are provided as a Source Data file.

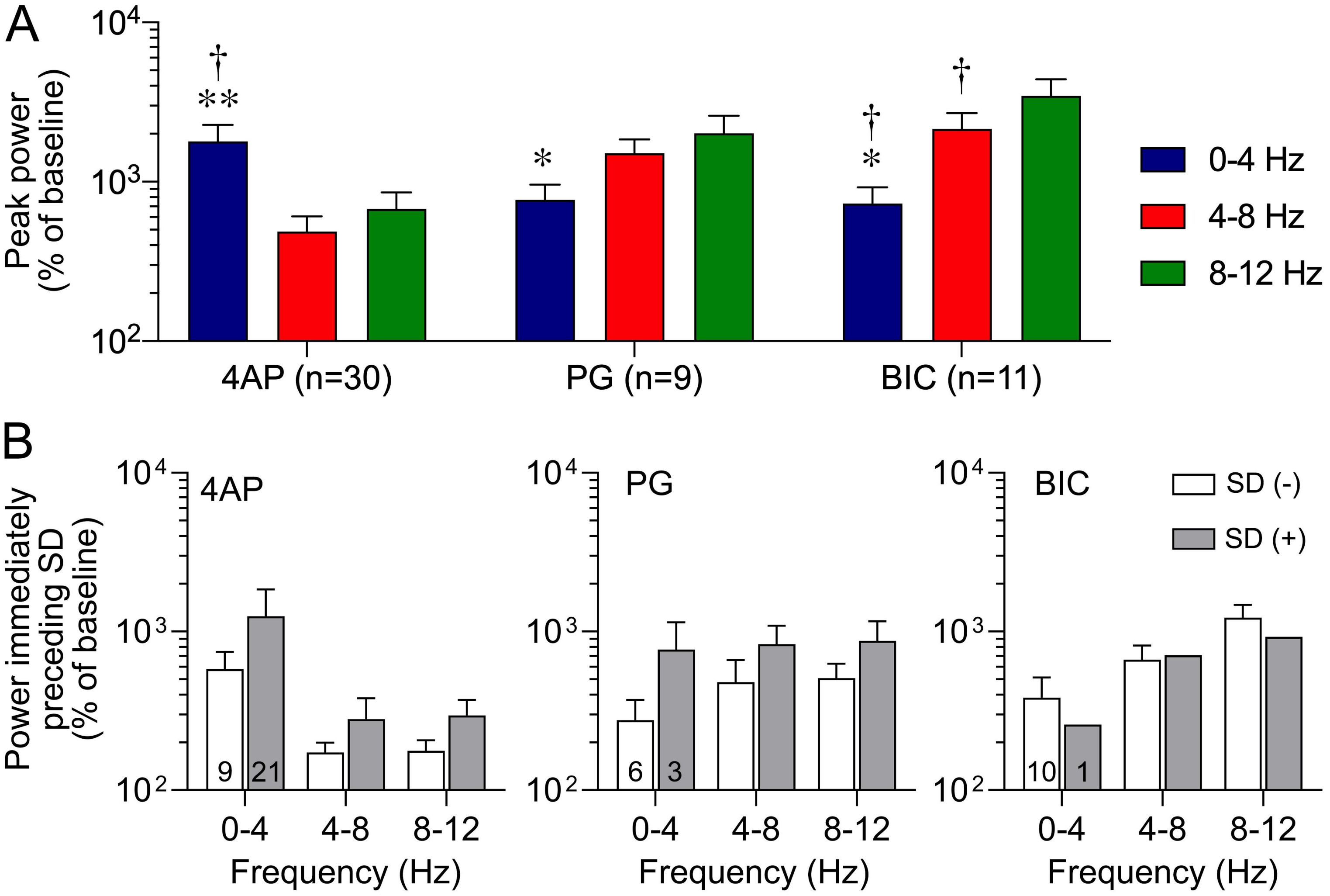

Supplementary Figure 4. Power density increase in the δ frequency range predicts SD occurrence.

(A) Peak power change during seizures induced by three different seizure-inducing agents are shown for each frequency band. To obtain the peak change, average power was calculated using FFT in 5-minute ECoG blocks and normalized to baseline for each experiment. The highest average power increase was then taken as peak change for each animal and averaged across the entire cohort. The δ frequency band (0-4 Hz) showed the largest power increase in 4AP-induced seizures where the highest rate of spontaneous SD occurred. *$p<0.05$ and **$p<0.01$ vs. 4-8 Hz; †$p<0.05$ vs. 8-12 Hz (two-way ANOVA for repeated measures). **(B)** This was supported by ECoG power increase immediately before the first SD compared with time-matched power increase in animals that did not develop an SD (5-minute averages normalized to baseline). Number of animals are indicated at the bottom of each bar. Data are mean±SEM. Source data are provided as a Source Data file.

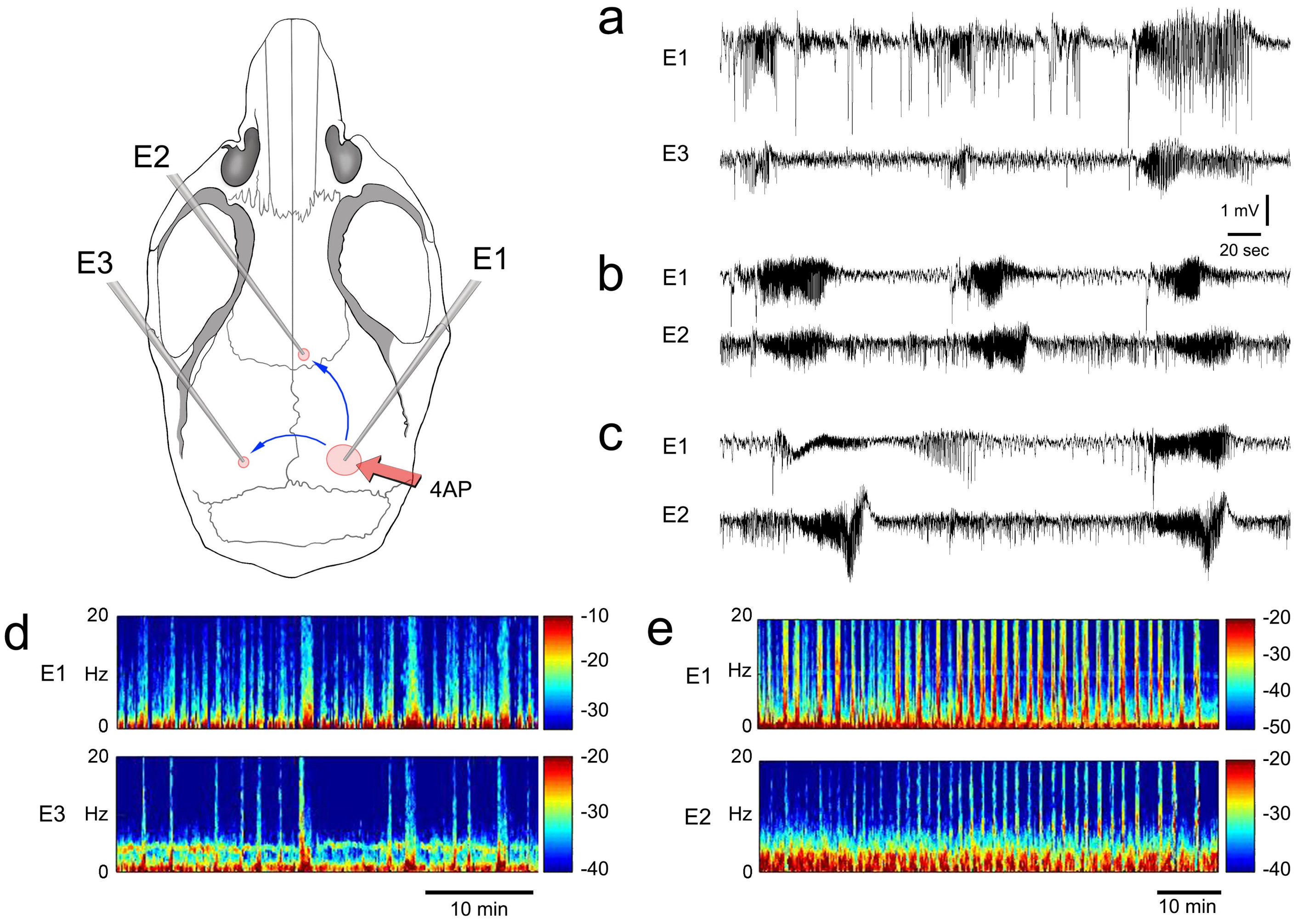

Supplementary Figure 5. Generalization of seizures across the cortex.

Experimental setup (upper left) to detect electrophysiological generalization of seizures (blue arrows) from the 4AP focus to the ipsilateral (E2) and contralateral (E3) remote electrodes. Representative ECoG tracings from different experiments show 4AP-induced seizure activity at the contralateral **(a)** and the ipsilateral **(b-c)** remote electrodes. Time-frequency spectra from two different experiments show synchronized power increases in contralateral **(d)** and ipsilateral **(e)** remote electrodes indicating seizure generalization. Source data are provided as a Source Data file.

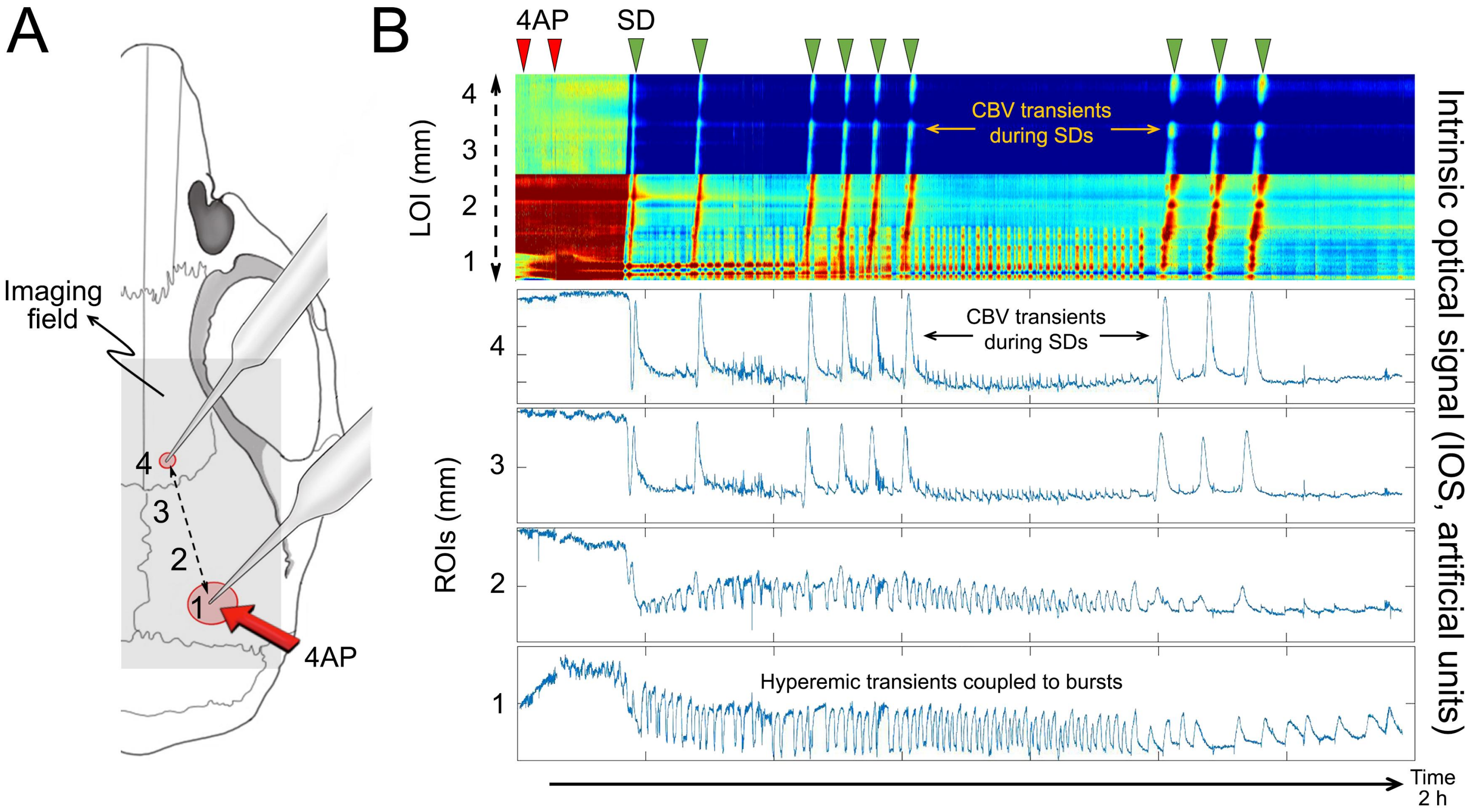

Supplementary Figure 6. Hemodynamic changes during seizure induced SDs.

(A) We placed a line of interest (LOI) extending from the 4AP application site anteriorly on the ipsilateral hemisphere. Four ROIs (1-4) were placed along the LOI at 1.2 mm intervals. **(B)** IOS intensity along the LOI and in each ROI are plotted over time. Red indicates increase and blue decrease in CBV. Recurrent SDs (green arrowheads) were associated with CBV changes typical of SDs in normal mouse cortex. The first SD triggered a triphasic response and all subsequent SDs were hyperemic, suggesting that cortex was not injured or hemodynamically compromised. The first SD penetrated ROIs 2-4 and partially affected ROI 1. Subsequent SDs did not penetrate ROI 1 (4AP application site) and had no effect on seizure-coupled hyperemic transients.Source data are provided as a Source Data file.

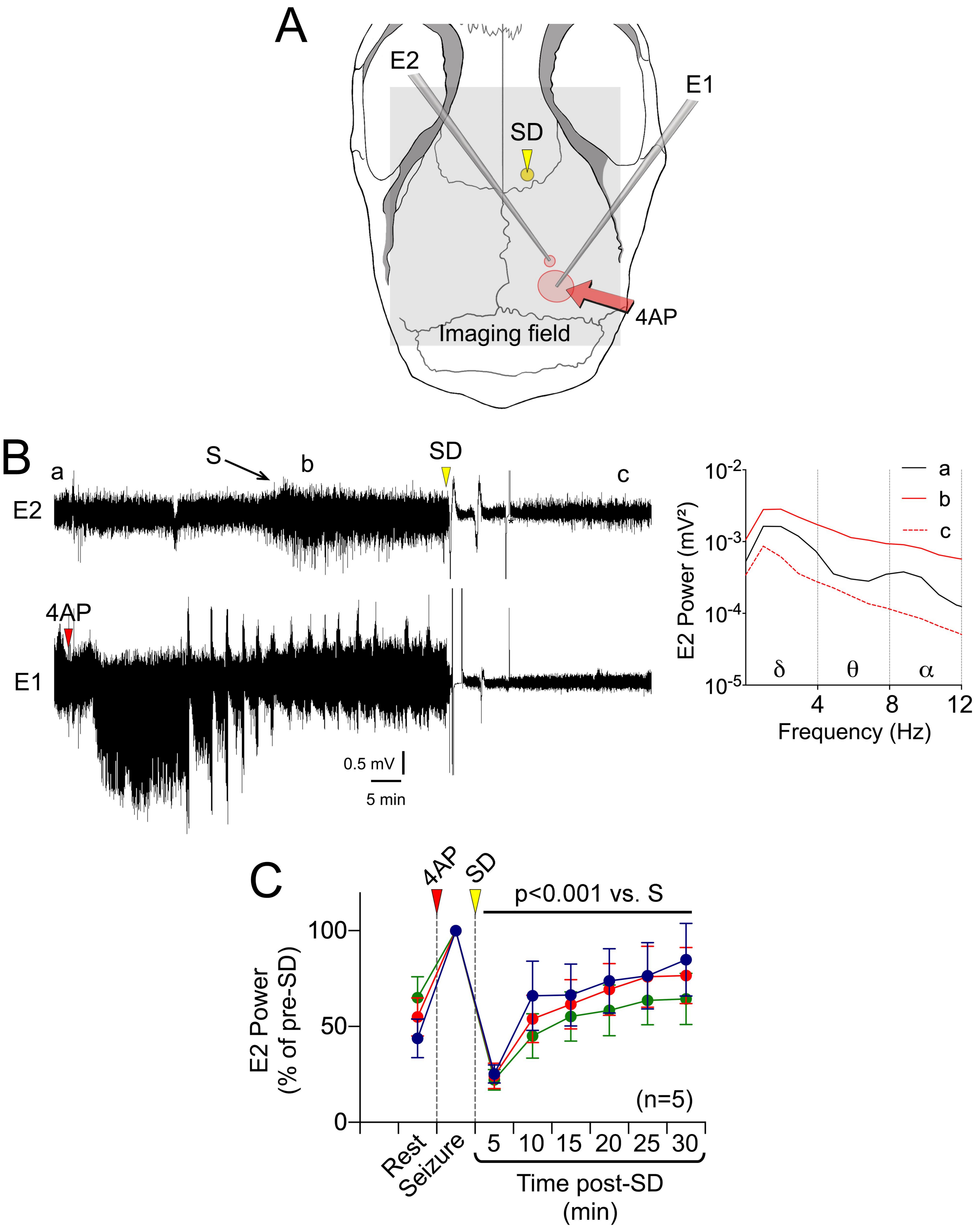

Supplementary Figure 7. SD limits spatial spread of seizures.

(A) Experimental setup showing two electrodes placed 1mm apart. An SD was induced via topical KCl application (yellow arrowhead) over the frontal cortex. **(B)** Representative ECoG tracing from E1 and E2 showing seizure starting 5 minutes after 4AP application in E1 and 35 minutes after 4AP application in E2 (S). SD was induced 20 minutes after ECoG power increased in E2, and immediately suppressed seizure activity. Power density computed using FFT at phases a-c are also shown. Recording in E1 briefly lost connection after SD induction creating an artefact. A second SD erupted 5 minutes after the first. **(C)** The ECoG power time course from E2 normalized to the pre-SD 5-minute average power shows the 4AP-induced increase in power from resting state (R) at this location outside the drug application site and significant reduction in seizure power after SD. ECoG power in E2 returned to baseline within 10 minutes but did not reach pre-SD seizure levels for at least 30 minutes ($p<0.001$ vs. pre-SD, one-way ANOVA; ±SEM). Source data are provided as a Source Data file.

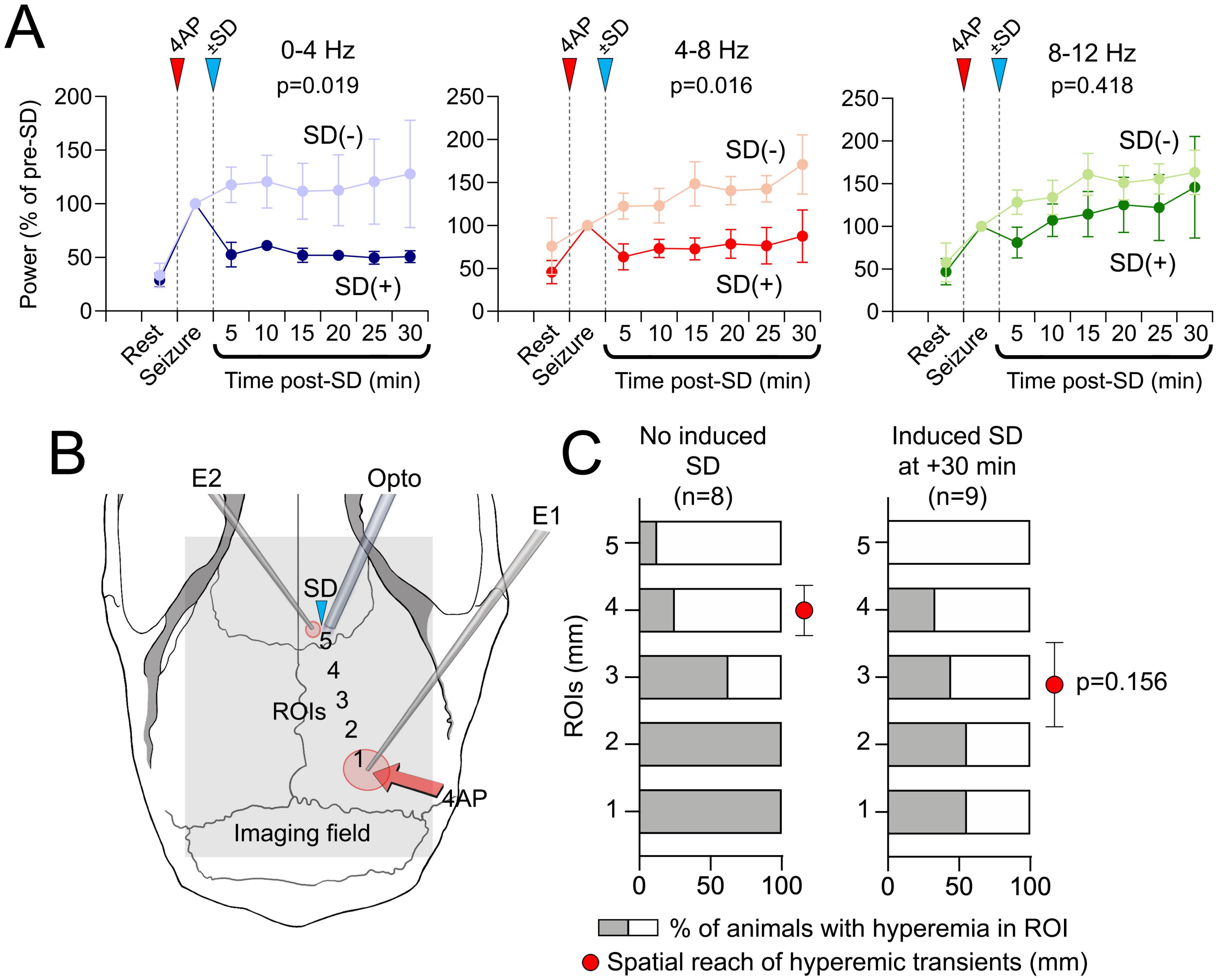

Supplementary Figure 8. Optogenetically-induced SDs are similarly antiepileptic.

(A) Averaged ECoG power time courses normalized to pre-SD (5 minutes) seizure power (S) from E1 are shown separately for each frequency band in ChR2+ mice. An optogenetic SD (blue arrowhead) induced at the remote site 29±3 minutes (range 19-40) after 4AP application significantly reduced seizure power (bold symbols) compared with time controls without SD (faint symbols, n=10 and 8, respectively). Exact p-values are shown on each graph (two-way ANOVA with 4AP-induced seizures are shown. **(B)** Experimental setup showing electrode (E1-E2), optogenetic light source (opto) and ROI (1-5) placement. **(C)** The proportion of animals that developed hyperemic transients coupled to 4AP seizures within each of the five ROIs and the average distance to which the hyperemia reached (red circles) are shown with and without an optogenetic SD (p=0.156, t-test with Welch's correction). Source data are provided as a Source Data file.

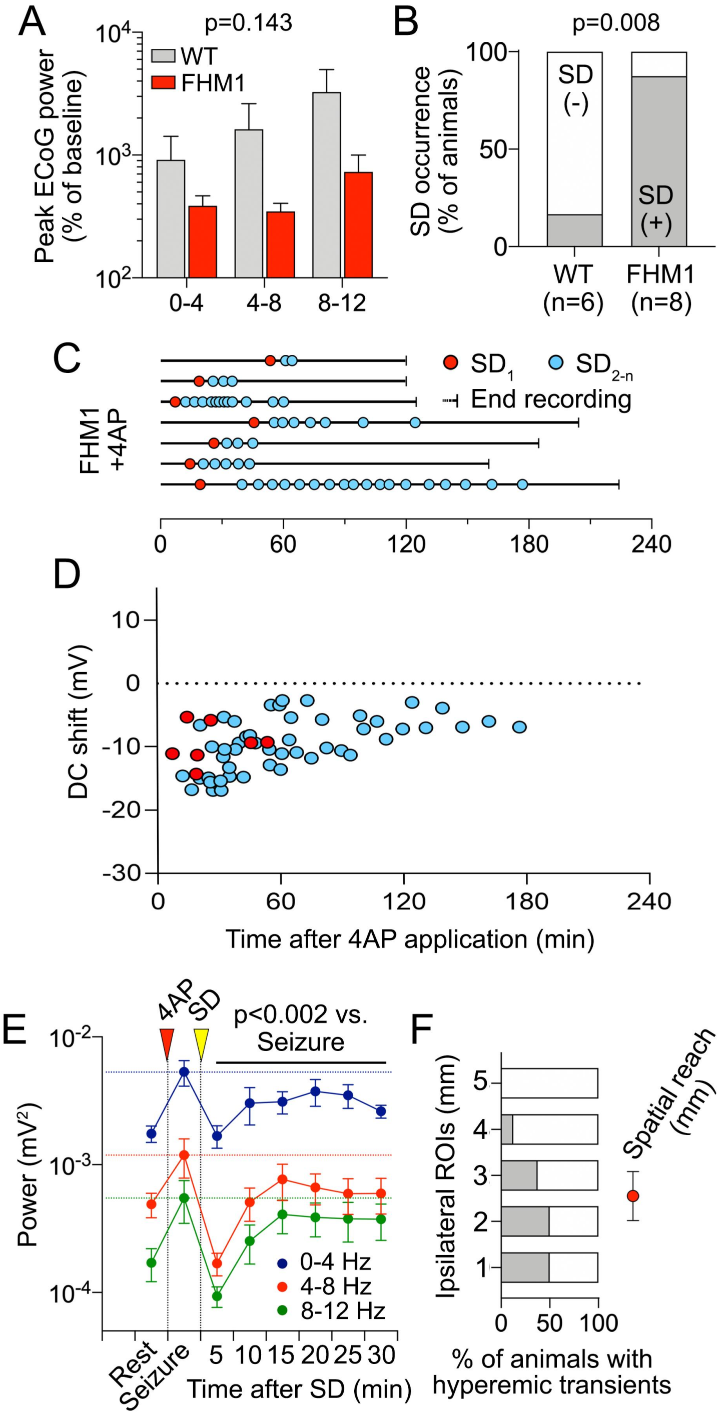

Supplementary Figure 9. Spontaneous SDs in familial hemiplegic migraine type 1 (FHM1) mutants and their antiepileptic effect.

(A) Peak power change during seizures normalized to baseline ECoG in FHM1 knockin mice and their WT littermates separated into frequency bands (p=0.143, 2-way ANOVA). **(B)** Fraction of animals that developed spontaneous SD from the seizure focus was significantly higher in FHM1 (p=0.008, χ^2). **(C)** Experimental timelines in FHM1 mutants that developed spontaneous SDs. Symbols represent first (SD1, red symbol) and subsequent SDs (SD2-n, blue symbol). FHM1 mutants developed a much higher frequency of SDs, which often occurred in clusters. **(D)** The change in DC shift amplitude of first and subsequent SDs over time in FHM1 mutant mice shows that, unlike WT mice shown in Figure 3, all but one SD penetrated the seizure focus (i.e. negative DC shift) even at late stages of the experiments**. (E)** Average ECoG power in the 4AP application site at rest (R), 4AP seizures 5 minutes before the first spontaneous SD (S), and every 5 minutes thereafter show that spontaneously occurring SDs exert an antiepileptic effect in FHM1 mutants (p=0.002 vs. pre-SD, 2-way ANOVA for repeated measures). **(F)** The proportion of animals that developed hyperemic transients coupled to 4AP seizures within each of the five ROIs and the average distance to which the hyperemia reached (red circles) in FHM1 mutants. Data are mean±SEM. Source data are provided as a Source Data file.

Supplementary Movie 1. IOS imaging of seizure activity and recurrent SDs.

A representative experiment is shown in which seizures were induced by 4AP-application (right parietal cortex). Frame rate is 1Hz. Time after initial 4AP application is indicated in seconds. Right panel shows unprocessed reflectance images in greyscale. The hyperemic response to seizure activity appears as decreased reflectance (i.e. darker) due to increase in total hemoglobin (i.e. CBV). Left panel shows CBV in arbitrary units calculated using MATLAB. Red indicates an increase, blue a decrease in CBV. Recurrent hyperemic transients lasting less than a minute each become visible in and around the focus before first SD occurs and gradually reach farther over time. A total of 9 SDs occur over approximately 2 hours starting 15 minutes after 4AP application. These hyperemic transients were coupled to seizure bursts. Images were acquired by a camera (MU300, AmScope, Irvine, CA, USA, Aptina MT9T001 CMOS sensor, 8-bit, resolution 2048x1536) attached to the microscope after skull preparation. Exposure time ranged from 200 to 350 ms and analog gain was kept under 2. Source data are provided as a Source Data file.

Supplementary Movie 2. Graphic depiction of SD as an endogenous antiseizure mechanism.

A unifying theory wherein SD is a fundamental endogenous antiseizure mechanism in the central nervous system. SD is triggered when intense synchronized focal neuronal network activity raises extracellular K^+ above the 12 mM threshold in a minimum critical volume of tissue estimated to be ~1 mm^3. SD then acts as an 'emergency brake' or 'reboot' extinguishing the seizure and propagates centimeters away from the focus to exert a broader antiseizure effect. The latter is clinically perceived as a migraine aura.

Supplementary Table 1. Systemic physiological parameters in animals with or without a spontaneous SD during 4AP seizures.

	**SD (-) (n=10)**	**SD (+) (n=21)**
**pH**	7.34 ± 0.02	7.35 ± 0.01
**pCO2** [mmHg]	34 ± 2.83	34 ± 1.08
**pO2** [mmHg]	137 ± 8.56	148 ± 4.46
**Gluc** [mg/dl]	149 ± 9.7	142 ± 5.46
**BP** [mmHg]	89 ± 4.15	94 ± 1.89

Data are mean ± SEM. Source data are provided as a Source Data file.
