# Supplementary material for: Spreading depression as an innate antiseizure mechanism
Source: Nat Commun. 2021 Apr 13;12:2206. doi: 10.1038/s41467-021-22464-x (PMC8044138; doi:10.1038/s41467-021-22464-x)
Supplement: Supplementary file 3 — Description of Additional Supplementary Files [file 41467_2021_22464_MOESM3_ESM.pdf]

Description of Additional Supplementary Files

Supplementary Movie 1: IOS imaging of seizure activity and recurrent SDs.

A representative experiment is shown in which seizures were induced by 4AP-application (right parietal cortex). Frame rate is 1Hz. Time after initial 4AP application is indicated in seconds. Right panel shows unprocessed reflectance images in greyscale. The hyperemic response to seizure activity appears as decreased reflectance (i.e. darker) due to increase in total hemoglobin (i.e. CBV). Left panel shows CBV in arbitrary units calculated using MATLAB. Red indicates an increase, blue a decrease in CBV. Recurrent hyperemic transients lasting less than a minute each become visible in and around the focus before first SD occurs and gradually reach farther over time. A total of 9 SDs occur over approximately 2 hours starting 15 minutes after 4AP application. These hyperemic transients were coupled to seizure bursts. Images were acquired by a camera (MU300, AmScope, Irvine, CA, USA, Aptina MT9T001 CMOS sensor, 8-bit, resolution 2048x1536) attached to the microscope after skull preparation. Exposure time ranged from 200 to 350 ms and analog gain was kept under 2.

Supplementary Movie 2: Graphic depiction of SD as an endogenous antiseizure mechanism.

A unifying theory wherein SD is a fundamental endogenous antiseizure mechanism in the central nervous system. SD is triggered when intense synchronized focal neuronal network activity raises extracellular K+ above the 12 mM threshold in a minimum critical volume of tissue estimated to be ~1 mm3. SD then acts as an ‘emergency brake’ or ‘reboot’ extinguishing the seizure and propagates centimeters away from the focus to exert a broader antiseizure effect. The latter is clinically perceived as a migraine aura.
