# Supplementary material for: Spreading depression as an innate antiseizure mechanism
Source: Nat Commun. 2021 Apr 13;12:2206. doi: 10.1038/s41467-021-22464-x (PMC8044138; doi:10.1038/s41467-021-22464-x)
Supplement: Supplementary file 6 — Reporting Summary [file 41467_2021_22464_MOESM6_ESM.pdf]

nature research

Corresponding author(s): Cenk Ayata, M.D.

Last updated by author(s): Feb 3, 2021

Reporting Summary

Nature Research wishes to improve the reproducibility of the work that we publish. This form provides structure for consistency and transparency in reporting. For further information on Nature Research policies, see our Editorial Policies and the Editorial Policy Checklist.

Statistics

For all statistical analyses, confirm that the following items are present in the figure legend, table legend, main text, or Methods section.

n/a	Confirmed	
[ ]	[x]	The exact sample size (*n*) for each experimental group/condition, given as a discrete number and unit of measurement
[ ]	[x]	A statement on whether measurements were taken from distinct samples or whether the same sample was measured repeatedly
[ ]	[x]	The statistical test(s) used AND whether they are one- or two-sided<br>*Only common tests should be described solely by name; describe more complex techniques in the Methods section.*
[ ]	[x]	A description of all covariates tested
[ ]	[x]	A description of any assumptions or corrections, such as tests of normality and adjustment for multiple comparisons
[ ]	[x]	A full description of the statistical parameters including central tendency (e.g. means) or other basic estimates (e.g. regression coefficient) AND variation (e.g. standard deviation) or associated estimates of uncertainty (e.g. confidence intervals)
[ ]	[x]	For null hypothesis testing, the test statistic (e.g. *F*, *t*, *r*) with confidence intervals, effect sizes, degrees of freedom and *P* value noted<br>*Give P values as exact values whenever suitable.*
[x]	[ ]	For Bayesian analysis, information on the choice of priors and Markov chain Monte Carlo settings
[x]	[ ]	For hierarchical and complex designs, identification of the appropriate level for tests and full reporting of outcomes
[x]	[ ]	Estimates of effect sizes (e.g. Cohen's *d*, Pearson's *r*), indicating how they were calculated

Our web collection on statistics for biologists contains articles on many of the points above.

Software and code

Policy information about availability of computer code

Data collection	LabChart 5 (ADInstruments, Colorado Springs, MO, USA) was used for aquisition of electrophysiological and blood pressure data. Labchart 8 Pro (ADInstruments, Colorado Springs, MO, USA) was used to calculate FFT of electrophysiological data and mean of blood pressure. Spreading depolarizations were optogenetically induced controlled by LabChart and an analog-digital converter (PowerLab, ADInstruments, Colorado Springs, CO, USA). YawCam 0.6.2 (2018) was used to capture full-field images of the skull for CBV calculation.
Data analysis	LabChart 5 (ADInstruments, Colorado Springs, MO, USA) was used for aquisition of electrophysiological and blood pressure data. Labchart 8 Pro (ADInstruments, Colorado Springs, MO, USA) was used to calculate FFT of electrophysiological data and mean of blood pressure. Time-varying spectrograms were computed via MATLAB (R2018b) using Thomson's multitaper method (Chronux toolbox, http://chronux.org; Chronux version 2.12 v03, 2018). An in-house MATLAB code was used to calculate CBV with the modified Beer-Lambert law as previously described (Ma, Y., et al., Wide-field optical mapping of neural activity and brain haemodynamics: considerations and novel approaches. Philos Trans R Soc Lond B Biol Sci, 2016. 371(1705).). Prism 8 (GraphPad Software) was used for statistical testing and generating graphs. The MATLAB code for calculating CBV changes using the modified Beer-Lambert law can be accessed through the following link: https://github.com/it078/CBV_Pixel_Analysis_Seizure_Spread.git

For manuscripts utilizing custom algorithms or software that are central to the research but not yet described in published literature, software must be made available to editors and reviewers. We strongly encourage code deposition in a community repository (e.g. GitHub). See the Nature Research guidelines for submitting code & software for further information.

Data

Policy information about availability of data

All manuscripts must include a data availability statement. This statement should provide the following information, where applicable:

- Accession codes, unique identifiers, or web links for publicly available datasets
- A list of figures that have associated raw data
- A description of any restrictions on data availability

The data that support the findings of this study are available from the corresponding author upon reasonable request.

Field-specific reporting

Please select the one below that is the best fit for your research. If you are not sure, read the appropriate sections before making your selection.

[x] Life sciences [ ] Behavioural & social sciences [ ] Ecological, evolutionary & environmental sciences

For a reference copy of the document with all sections, see nature.com/documents/nr-reporting-summary-flat.pdf

Life sciences study design

All studies must disclose on these points even when the disclosure is negative.

Sample size	A total of 197 mice were included in the study. 16 mice were excluded (see below). In the absence of prior experience, sample sizes were selected empirically to at least detect a 30% effect size based on a coefficient of variation of 20% (alpha=0.05, beta=0.8).
Data exclusions	N=4 experiments were excluded for technical problems (Amplifier on Powerlab did not measure DC amplitudes continuously due to software connection problems)<br>N=7 experiments were excluded because SDs were induced and captured via optical imaging during surgical preparation (2 FHM1 mice, 5 CD1 mice in spontaneous SD cohort, 4AP, PG)<br>N=1 experiment was excluded because the animal died (FHM1 mutant mouse due to severe seizures)<br>N=4 experiments were excluded because of altered drug actvity (Pre-treatment of cortex with SD, CD1 mice-cohort, 5)
Replication	Reproducibility of the experimental findings was verified by studying multiple cohorts at different points in time. Control experiments (e.g., no intervention vs. SD as a pre-treatment) were always conducted in parallel and randomized on the experimental day by an independent lab member. Experiments of cohorts (e.g. spontaneous SD occurrence during seizures) were replicated at least twice during the whole study at different time points (interval six months). In addition, fresh drug batches were obtained every three to four months to ensure pharmacological stability. This accounts for all cohorts described in the results section in the manuscript. All attempts at replication were successful.
Randomization	SD induction (yes/no) was randomized by an independent lab member after completion of surgical procedures.
Blinding	Real-time electrophysiological recordings and IOS images precluded blinding during data collection. Original analyses were confirmed in a blinded fashion.

Reporting for specific materials, systems and methods

We require information from authors about some types of materials, experimental systems and methods used in many studies. Here, indicate whether each material, system or method listed is relevant to your study. If you are not sure if a list item applies to your research, read the appropriate section before selecting a response.

Materials & experimental systems

n/a	Involved in the study	
[x]	[ ]	Antibodies
[x]	[ ]	Eukaryotic cell lines
[x]	[ ]	Palaeontology and archaeology
[ ]	[x]	Animals and other organisms
[x]	[ ]	Human research participants
[x]	[ ]	Clinical data
[x]	[ ]	Dual use research of concern

Methods

n/a	Involved in the study	
[x]	[ ]	ChIP-seq
[x]	[ ]	Flow cytometry
[x]	[ ]	MRI-based neuroimaging

Animals and other organisms

Policy information about studies involving animals; ARRIVE guidelines recommended for reporting animal research

Laboratory animals	- Wild-type mice (n=165, CD1, male, 3.2 +/- 0.1 months , Charles River Laboratories, Wilmington, MA, USA)<br>- Familial hemiplegic migraine type 1 (FHM1) knockin mice heterozygous for the S218L mutation in the mouse Cacna1a gene encoding for the alpha1A pore-forming subunit of CaV2.1 voltage-gated calcium channels (n=7 males, 1 female; 8.9+/-2.7 months)<br>- FHM1 wild type littermates (n=4 males, 2 females; 8.3+/-1.6 months)<br>- Transgenic mice expressing channelrhodopsin-2 (ChR2+, n=10 males and 8 females, 7.5+/-0.6 months, B6.Cg-Tg(Thy1-COP4/EYFP) 18Gfng/J, Jackson Laboratories, Bar Harbor, ME, USA)<br>The majority of mice were fasted overnight (17.4+/- 0.3 hours; n=173), while a small cohort was studied without fasting (n=17). Room temperature of the animal facility was approximately 25°C. The relative humidity was kept at 45 to 65%. Lights were turned of at 7 pm. and turned on at 7 am. providing a 12-hour light-dark cycle. Animals were kept in one cage in groups of 2-4.
Wild animals	No wild animals were used in the study.
Field-collected samples	No field collected samples were used in the study.
Ethics oversight	All experiments were approved by the Massachusetts General Hospital Institutional Animal Care and Use Committees and followed the NIH Guide for Use and Care of Laboratory Animals (NIH Publication No. 85-23, 1996).

Note that full information on the approval of the study protocol must also be provided in the manuscript.
