# Supplementary figures and images for: Spreading depression as an innate antiseizure mechanism
Source: Nat Commun. 2021 Apr 13;12:2206. doi: 10.1038/s41467-021-22464-x (PMC8044138; doi:10.1038/s41467-021-22464-x)

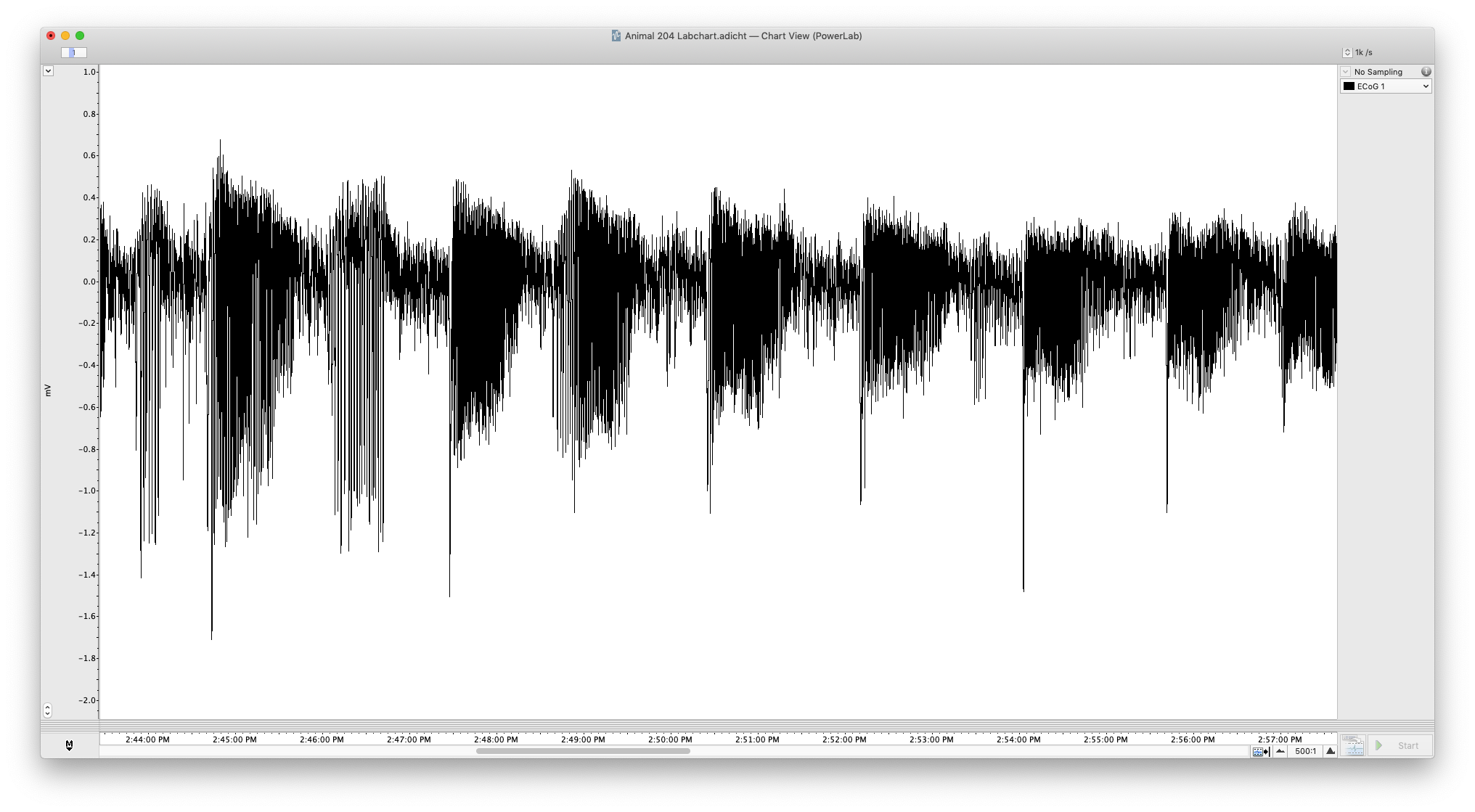

Supplement: Supplementary file 7 — Source Data [file 41467_2021_22464_MOESM7_ESM.zip › Source Data Excel 20210303/Figures/Figure 4/Fig. 4 Panel A/Screen Shot 2019-11-23 at 5.00.52 AM.png]
